# DedA Protein Relates to Action-Mechanism of Halicyclamine A, a Marine Spongean Macrocyclic Alkaloid, as an Anti-dormant Mycobacterial Substance

**DOI:** 10.3390/md9060984

**Published:** 2011-06-08

**Authors:** Masayoshi Arai, Liu Liu, Takao Fujimoto, Andi Setiawan, Motomasa Kobayashi

**Affiliations:** 1 Graduate School of Pharmaceutical Sciences, Osaka University, Yamada-oka 1-6, Suita, Osaka 565-0871, Japan; E-Mails: liuliu_cpu@hotmail.com (L.L.); ph5065ft@ecs.cmc.osaka-u.ac.jp (T.F.); 2 Department of Chemistry, Faculty of Science, Lampung University, Jl. Prof. Dr. Sumantri Brodjonegoro No. 1, Bandar Lampung 35145, Indonesia; E-Mail: setiawan_a22@yahoo.com (A.S.)

**Keywords:** halicyclamine A, dedA, marine sponge, dormant, tuberculosis

## Abstract

A macrocyclic alkaloid, halicyclamine A, was re-discovered from an Indonesian marine sponge of *Haliclona* sp. 05A08 as an anti-dormant mycobacterial substance. To clarify action-mechanism of halicyclamine A, halicyclamine A-resistant strains were screened from the transformants of *Mycobacterium smegmatis* with the genomic DNA library of *M. bovis* BCG, which were constructed in the multi-copy shuttle cosmid pYUB145. Sequencing analysis of the cosmids isolated from the halicyclamine A-resistant transformants revealed that the responsible gene was involved in the genome region between 2920.549 kb and 2933.210 kb. Further experiments using the transformants over-expressing individual gene contained in the responsible region were executed, and the transformant, which over-expressed *BCG2664* gene assigned as *dedA* gene, was found to become halicyclamine A-resistant. This evidence strongly suggested that DedA protein correlates with the action-mechanism of halicyclamine A as an anti-dormant mycobacterial substance.

## 1. Introduction

**Figure 1:**
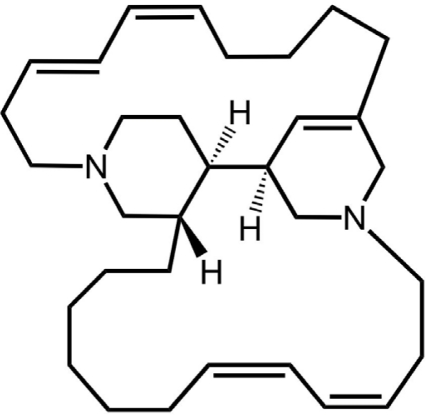
Chemical structure of halicyclamine A.

Tuberculosis (TB) is one of the most common causes of morbidity and mortality in HIV-positive adults living in poverty [1]. Eight million new TB cases and two million deaths from TB are estimated each year [2]. It is now generally accepted that the requirement for a minimum six months treatment for TB is due to the difficulty in eradicating non-replicating persistent *Mycobacterium tuberculosis*. Therefore, new lead compounds, which are effective against *M. tuberculosis* in both active and dormant states, are urgently needed. Although physiology of latent *M. tuberculosis* infection is still unclear, hypoxic condition was found to induce dormant state of *Mycobacterium* sp., which has a drug susceptibility profile resembling that of the latent *M. tuberculosis* infection [3–5]. Recently, we have established a screening system searching for anti-dormant mycobacterial substances [6–8]. In this assay system, *M. tuberculosis* H37Ra under hypoxic conditions, became highly resistant against isoniazid, which is one of the first-line drugs for TB. Thus, the MIC value of isoniazid against *M. tuberculosis* H37Ra was 0.05 μg/mL under standard aerobic growth condition. While, the MIC value of isoniazid was more than 100 μg/mL under nitrogen atmosphere containing 0.2% oxygen. In the course of our search for anti-dormant mycobacterial substances from marine organisms by using this assay system, a macrocyclic alkaloid, halicyclamine A (Figure 1), has been re-discovered as an anti-dormant Mycobacterial substance from an Indonesian marine sponge of *Haliclona* sp 05A08 [6]. Halicyclamine A showed potent anti-microbial activity against *M. smegmatis* (MIC = 2.5 μg/mL), *M. bovis* BCG (MIC = 1.0 μg/mL), and *M. tuberculosis* H37Ra (MIC = 5.0 μg/mL) under standard aerobic growth condition as well as dormancy-inducing hypoxic condition [6]. On the other hand, M. Jaspars, *et al.* reported that the crude spongean extract containing halicyclamine A showed an inhibitory activity against human inosine 5’-monophosphate dehydrogenase (IMPDH) [9]. In order to clarify action-mechanism of halicyclamine A, we previously prepared the transformants of *M. smegmatis*, which over-expressed mycobacterial IMPDHs, since the transformant over-expressing target proteins often confer drug-resistance [10,11]. However, the IMPDH over-expressing strains showed the same MIC value against halicyclamine A with that of the wild-type *M. smegmatis* [6]. In this paper, our continued study of the action-mechanism of halicyclamine A is presented.

## 2. Results and Discussion

### 2.1. Isolation of the Halicyclamine A-Resistant Clones from the Transformants of *M. smegmatis* with Genomic DNA Library

**Figure 2:**
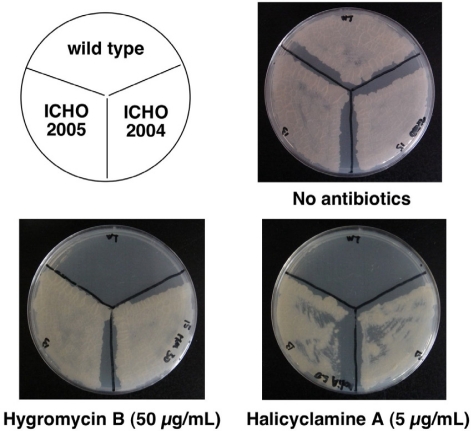
Halicyclamine A resistant-strains of *M. smegmatis* transformed with genomic DNA library. The wild-type strain and transformants (ICHO2004 and ICHO2005) of *M. smegmatis* were cultured in 7H9 broth, and the concentration of the each culture was adjusted to 1 × 10^5^ CFU/mL. The 10 μL aliquot of each culture was spread on a 7H10 agar plate in the presence or absence of halicyclamine A (5.0 μg/mL).

**Figure 3:**
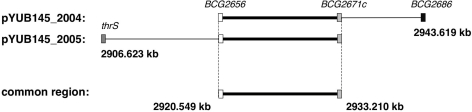
Gene maps of the cosmids extracted from the halicyclamine A-resistant transformants.

To identify the gene that could confer a resistance to halicyclamine A, we prepared transformants of *Mycobacterium smegmatis*, which were transformed with the genomic DNA library of *M. bovis* BCG constructed in the multi-copy shuttle cosmid pYUB145. Then, halicyclamine A-resistant clones were screened from over the 4,000 transformants by the dish culture containing 5.0 μg/mL (2 × MIC value for *M. smegmatis*) of halicyclamine A. We could isolate two transformants, designated ICHO2004 and ICHO2005, as the resistant strains to halicyclamine A (Figure 2). Each resistant strain was cultured in the Middlebrook 7H9 broth containing hygromycin B, and the two cosmids (pYUB145_2004 and pYUB145_2005) were extracted from the ICHO2004 strain and ICHO2005 strain, respectively. Then, the pYUB145_2004 and pYUB145_2005 cosmids were found to contain 23.070 kb (coordinates from 2920.549 kb to 2943.619 kb) and 26.587 kb (coordinates from 2906.623 kb to 2933.210 kb) genome fragments, respectively. This data indicated that the gene for giving resistnace to halicyclamine A would be contained in the genome region of *M. bovis* BCG from 2920.549 kb to 2933.210 kb (12.661 kb) (Figure 3).

### 2.2. Second Selection of the Transformants of *M. smegmatis* Expressing the Candidate Region Showing Resistance to Halicyclamine A

**Figure 4:**
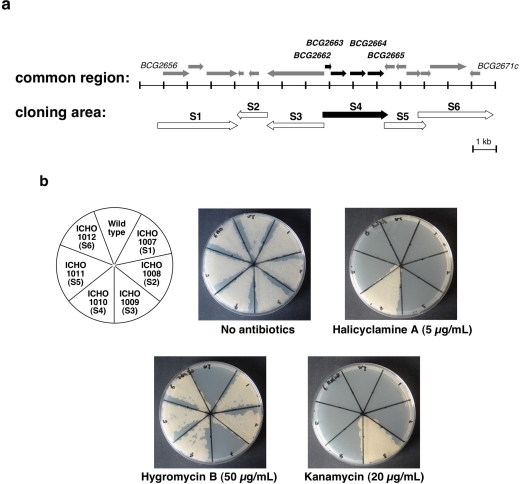
Growth of the transformants of *M. smegmatis* over-expressing candidate area showing resistance to halicyclamine A on the 7H10 agar plate containing halicyclamine A. (**a**) Gene map of the cloning area to prepare transformants of *M. smegmatis*. (**b**) Each strain (wild-type of *M. smegmatis*, ICHO1007, ICHO1008, ICHO1009, ICHO1010, ICHO1011 and ICHO1012) was cultured in the 7H9 broth, and then the cultures were adjusted to 1 × 10^5^ CFU/mL. The 10 μL aliquots of each culture were spread on the 7H10 agar plates in the presence or absence of halicyclamine A (5.0 μg/mL).

To narrow down candidate genes expressing resistance to halicyclamine A, we then divided the 12.661 kb common region into six areas (S1–S6) as shown in Figure 4a and established six transformants (ICHO1007–ICHO1012) of *M. smegmatis*, which over-expressed each area. As shown in Figure 4b, all established transformants showed resistance against hygromycin B or kanamycin, which corresponds to the antibiotic-resistance genes in the plasmids of pMV206 or pMV261. The transformant of ICHO1010, which over-expressed the S4 area containing *BCG2662*, *BCG2663*, *BCG2664* and *BCG2665* genes, showed resistance against 5.0 μg/mL concentartion of halicyclamine A, whereas no resistance to halicyclamine A was observed in both wild-type and other transformants of *M. smegmatis.*

### 2.3. Identification of the Gene Causing Resistance to Halicyclamine A

**Figure 5:**
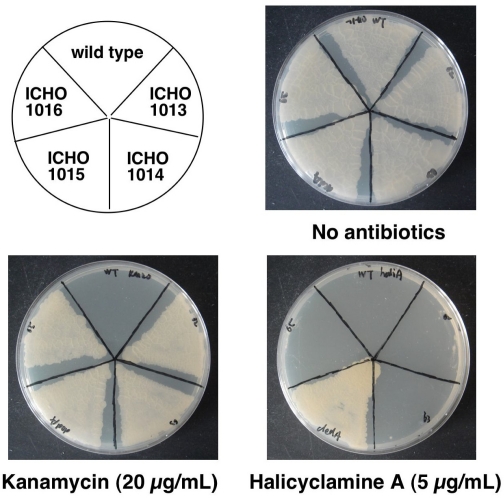
Growth of the transformants of *M. smegmatis* over-expressing the candidate gene for giving resistance to halicyclamine A on the 7H10 agar plate containing halicyclamine A. Each strain (wild-type of *M. smegmatis*, ICHO1013, ICHO1014, ICHO1015 and ICHO1016) was cultured in the 7H9 broth, and then the cultures were adjusted to 1 × 10^5^ CFU/mL. The 10 μL aliquots of each culture were spread on the 7H10 agar plate in the presence or absence of halicyclamine A (5.0 μg/mL).

To identify the gene causing resistance to halicyclamine A, four transformants (ICHO1013–ICHO1016) of *M. smegmatis*, which over-expressed each *BCG2662*, *BCG2663*, *BCG2664* and *BCG2665* gene, were prepared, and we observed growth of the four transformants on the 7H10 agar containing 5 μg/mL concentration of halicyclamine A. As a result, the only ICHO1015 strain, which over-expresses *BCG2664* gene assigned as *dedA* gene, showed resistance to halicyclamine A (Figure 5). In addition, the MIC value of halicyclamine A against ICHO1015 strain was evaluated 5.0 μg/mL by agar dilution assay using 7H10 agar plate, whereas 2.5 μg/mL of halicyclamine A inhibited the growth of the wild type of *M. smegmatis* completely (data not shown) [12]. Although further study is necessary for the identification of the target molecule of halicyclamine A, these results strongly suggested that DedA protein correlates with the action mechanism of halicyclamine A as an anti-dormant mycobacterial substance.

DedA family gene exists in most bacterial genome and is speculated to encode integral membrane protein with completely unknown functions. *M. smegmatis*, *M. bovis* BCG and *M. tuberculosis* H37Rv have seven genes of *dedA* family in their genome, respectively. The homology between *BCG2664* gene and one of *dedA* genes in *M. tuberculosis* H37Rv is 100%, while the homology with that in *M.smegmatis* is 38%. Although no precise function of DedA family protein has been reported, several studies suggested that DedA family protein might function as envelope biogenesis and maintenance. For instance, a DedA protein in *Ralstonia metallidurans* was shown to be required for resistance to selenite and was thought to be involved in transport function [13]. The *yghB* and *yqjA* genes, which belong to the *dedA* gene family, play redundant and critical roles in membrane biology that are essential for completion of cell division in *E. coli* [14]. From these observations, halicyclamine A possibly inhibits the function of envelope biogenesis and maintenance in *Mycobacterium* species by inhibition of DedA protein.

## 3. Experimental Section

### 3.1. Materials

Middlebrook 7H9 broth, Middlebrook 7H10 agar, Middlebrook OADC Enrichment, and Luria-Bertani (LB) broth were obtained from BD (Franklin Lakes, NJ, USA). DNA restriction enzymes and T4 DNA ligase were obtained from New England BioLabs INC. (Ipswich, MA, USA). Expand High Fidelity PCR System (Roche Applied Science, Mannheim, Germany) was used for PCR. Carbenicillin, hygromycin B, isoniazid, kanamycin and other chemicals were purchased from Sigma (St. Louis, MO, USA). Halicyclamine A was isolated from Indonesian marine sponge of *Haliclona* sp. 05A08 (Figure 1). Briefly, the methanol extract (43 g) of the dry sponge (200 g) were partitioned by the alkaloid extraction procedure. The alkaloid fraction (2.5 g) was subjected to silica gel column chromatography (eluted with CHCl_3_-MeOH containing 1.0% triethylamine) and HPLC [COSMOSIL Sugar-D column (Nacalai tesque, Kyoto, Japan); eluted with CHCl_3_-CH_3_CN-H_2_O] to isolate halicyclamine A (20% yield from the alkaloid fraction). Halicyclamine A was identified by ESI-TOF-MS and 2D-NMR analyses and comparison with authentic spectral data [9,15].

### 3.2. Bacterial Culture

*Mycobacterium* strains were grown in Middlebrook 7H9 broth containing 10% Middlebrook OADC enrichment, 0.2% glycerol, and 0.05% Tween 80, or on Middlebrook 7H10 agar containing 10% Middlebrook OADC enrichment and 0.5% glycerol. *Escherichia coli* DH5α was used for cloning and maintaining plasmid and grown in Luria-Bertani (LB) liquid medium. *E. coli* HB101 was used for construction of the genomic DNA library of *M. bovis* BCG and grown in LB liquid medium containing 0.3% maltose and 10 mM MgSO_4_. The concentrations of antibiotics used were 100 μg/mL (carbenicillin), 150 μg/mL (hygromycin B), and 40 μg/mL (kanamycin) for *E. coli* strains and 50 μg/mL (hygromycin B) and 20 μg/mL (kanamycin) for *Mycobacterium* strains, respectively.

### 3.3. Construction of Gnomic DNA Library and Transformation of *M. smegmatis* mc^2^155

The chromosomal DNA of *M. bovis* BCG Pasteur was prepared by the hexadecyltrimethylammonium bromide (CTAB) method [16]. The chromosomal DNA was then digested with restriction endonuclase *Sau3AI* to produce approximately 30 kb DNA fragments. Genomic DNA library was constructed in the *E. coli*-*Mycobacterium* shuttle cosmid, pYUB145, by using the double *cos* vector strategy as previously described [17]. Briefly, the left and right arms of pYUB145 were generated by digestion with restriction endonuclases of *XbaI* and *BamHI*. The fragments of genome were ligated to the both arms of pYUB145. The mixture of ligations were *in vitro* packaged with packaging mix of MaxPlax Lambda Packaging (Epicentre), and the resulting recombinant cosmids were transduced to *E. coli* HB101. The transformants of *E. coli* HB101 were selected on the LB agar plates containing carbenicillin. Over 3 × 10^5^ independent clones were pooled, and cosmids for transformation of *M. smegmatis* was obtained by large scale DNA preparation using a standard alkaline lysis method. In order to prepare transformants of *M. smegmatis* with genomic DNA library, *M. smegmatis* mc^2^155 were grown at 37 °C as described above until the optical density reached 0.8–1.0 at 600 nm. The cultures were centrifuged, and the resulting pellets were washed with 10% glycerol twice and re-suspended in the same solution (1/10th of the initial culture volume). The cell suspensions were mixed with gnomic DNA library and electroporated (2500 V, 25 μF, 1000 Ω). The resulting suspensions were incubated at 37 °C for 4 h and then plated on Middlebrook 7H10 agar containing 50 μg/mL of hygromycin B.

### 3.4. Isolation of Halicyclamine A-Resistant Clones from the Transformants of *M. smegmatis* with Genomic DNA Library and End Sequencing of Cosmids

Halicyclamine A-resistant clones were screened from over the 4 × 10^3^ transformants of *M. smegmatis* with the genomic DNA library by cultivating on the 7H10 agar containing 5.0 μg/mL concentration of halicyclamine A. Then, the resistant clones against halicyclamine A were grown in the 7H9 broth containing hygromycin B, and cosmids for end sequencing were isolated by using standard alkaline lysis method. The cosmids extracted from the halicyclamine A-resistant transformants were subjected to end sequencing (MACROGEN, South Korea). The primers P1 (5’-GTACGCCACCGCCTGGTTC-3’) and P2 (5′-GTGCCACCTGACGTCTAAG-3’), which were designed based on the sequence of cosmid vector pYUB415, were used to amplify the integrated end sequences of the cosmids. The obtained sequences were analyzed by BLAST search using the database of BCGList (http://genolist.pasteur.fr/BCGList/) and Comprehensive Microbial Resource in J. Craig Venter Institute (http://cmr.tigr.org/cgi-bin/CMR/CmrHomePage.cgi).

### 3.5. Preparation of the Transformants of *M. smegmatis* Over-Expressing the Candidate-Genes for Giving Resistance to Halicyclamine A

The gene regions or genes for candidate giving resistance to halicyclamine A were PCR amplified from the cosmids pYUB415_2004 or pYUB415_2005 by using the primer pairs as shown in Table 1. PCR was performed using a program of 30 cycles of 94 °C for 15 sec, 57 °C for 30 sec and 68 °C for 1 min/kb. Following cloning into pCR2.1-TOPO (Invitrogen) and sequencing, the cloned PCR fragment was excised using the primer-introduced restriction sites and cloned into the mycobacterial expression vector pMV261 having *hsp60* promoter and kanamycin-resistant gene or promoter-less shuttle vector pMV206 having hygromycin B-resistant gene. Description of plasmids and name of transformants were listed as shown in Table 1. The transformation of *M. smegmatis* was performed by above mentioned method.

## 4. Conclusions

We previously found that a marine spongian macrocyclic alkaloid, halicyclamine A, showed potent anti-mycobacterial activity under standard aerobic growth conditions as well as dormancy-inducing hypoxic condition, although its action-mechanism was not clear. In this paper, we searched the gene, which gives a resistance to halicyclamine A, using the genomic DNA library of *M. bovis* BCG, since the transformants over-expressing target proteins often confer drug-resistance. Then, we finally found that a transformant of *M. smegmatis*, which over-expressed *BCG2664* gene coded DedA protein, showed the resistance to halicyclamine A. This evidence strongly suggested that DedA protein correlates with the action-mechanism of halicyclamine A as an anti-dormant Mycobacterial substance.

## Figures and Tables

**Table 1:** List of the transformants of *M. smegmatis* and description of plasmids.

**Strains**	**Plasmids**	**Description of plasmids**	**Sequence of primers (5’ to 3’)** F; Forward primer, R; Reverse primer
ICHO1007	pMV206	Cloned S1 area^#^ (2919991–2923343 bp of *M. bovis* BCG genome)*	F:TCTAGAGCCACACCCTGATAGCATTG R: AAGCTTTCGCGACGGCTGGAATG
ICHO1008	pMV206	Cloned S2 area ^#^ (2924490–2923316 bp of *M. bovis* BCG genome)*	F:GGATCCAACTGTTTCCGCACGAGGAG R:CTGCAGTCAACGGCGAACATTCCAGC
ICHO1009	pMV261	Cloned S3 area ^#^ (2926966–2924589 bp of *M. bovis* BCG genome)*	F:GGATCCATCCAGGAGGTCGCAGATGTC R: GAATTCGTGATGTTCGCGCCTAACGG
ICHO1010	pMV206	Cloned S4 area ^#^ (2926766–2929363 bp of *M. bovis* BCG genome)*	F:CAGCTGATAGCTCTGCGCGTACGAC R: TTCGAAGATCAGCAACGGGCTGGAC
ICHO1011	pMV206	Cloned S5 area ^#^ (2929454–2931112 bp of *M. bovis* BCG genome)*	F: GGATCCTGCGGTATCGCTTGCCTTG R: AAGCTTGCGCGACTACGCAGCTC
ICHO1012	pMV206	Cloned S6 area ^#^ (2930898–2933532 bp of *M. bovis* BCG genome)*	F:GGATCCAAGCGGCTAACTGTAGGCCTG R: AAGCTTTCAGCCCGGTGGTGTTGG
ICHO1013	pMV261	Cloned *BCG2662* gene (2926980–2927222 bp of *M. bovis* BCGgenome)*	F:GAATTCAGGACAACCCGCATGGTCG R: AAGCTTTATCACTCAGCGCCGGTACG
ICHO1014	pMV261	Cloned *BCG2663* gene (2927223–2927900 bp of *M. bovis* BCG genome)*	F: GAATTCCCGGTCAGGCATTCTTTACCC R:AAGCTTGAAAGTTCCAGTTGCGCACGAC
ICHO1015	pMV261	Cloned *BCG2664* gene(2928096–2928752 bp of *M. bovis* BCG genome)*	F: GAATTCCCGGTCAGGCATTCTTTACCC R: AAGCTTCAAGCCGGTCGGTCACG
ICHO1016	pMV261	Cloned *BCG2665* gene(2928915–2929361 bp of *M. bovis* BCG genome)*	F: GGATCCATGCACCATGGGCCTGATCAC R: GAATTCTACGGCCGGGATCAGCAAC

#; Name of area corresponds to the Figure 4a.

*; Coordinate of *M. bovis* BCG genome is based on the database of BCGList (http://genolist.pasteur.fr/BCGList/).
